# Contributions of biarticular myogenic components to the limitation of the range of motion after immobilization of rat knee joint

**DOI:** 10.1186/1471-2474-15-224

**Published:** 2014-07-07

**Authors:** Momoko Nagai, Tomoki Aoyama, Akira Ito, Hirotaka Iijima, Shoki Yamaguchi, Junichi Tajino, Xiangkai Zhang, Haruhiko Akiyama, Hiroshi Kuroki

**Affiliations:** 1 Department of Motor Function Analysis, Human Health Sciences, Graduate School of Medicine, Kyoto University, 53 Shogoin, Kawahara-cho, Sakyo-ku, Kyoto 606-8507, Japan; 2 Department of Development and Rehabilitation of Motor Function, Human Health Sciences, Graduate School of Medicine, Kyoto University, Kyoto, Japan; 3 Department of Orthopaedic Surgery, Graduate School of Medicine, Gifu University, Gifu, Japan

**Keywords:** Contracture, External fixators, Muscles, Range of motion, Rats

## Abstract

**Background:**

Muscle atrophy caused by immobilization in the shortened position is characterized by a decrease in the size or cross-sectional area (CSA) of myofibers and decreased muscle length. Few studies have addressed the relationship between limitation of the range of motion (ROM) and the changes in CSA specifically in biarticular muscles after atrophy because of immobilization. We aimed to determine the contribution of 2 distinct muscle groups, the biarticular muscles of the post thigh (PT) and those of the post leg (PL), to the limitation of ROM as well as changes in the myofiber CSAs after joint immobilization surgery.

**Methods:**

Male Wistar rats (n = 40) were randomly divided into experimental and control groups. In the experimental group, the left knee was surgically immobilized by external fixation for 1, 2, 4, 8, or 16 weeks (n = 5 each) and sham surgery was performed on the right knee. The rats in the control groups (n = 3 per time point) did not undergo surgery. After the indicated immobilization periods, myotomy of the PT or PL biarticular muscles was performed and the ROM was measured. The hamstrings and gastrocnemius muscles from the animals operated for 1 or 16 weeks were subjected to morphological analysis.

**Results:**

In immobilized knees, the relative contribution of the PT biarticular myogenic components to the total restriction reached 80% throughout the first 4 weeks and decreased thereafter. The relative contribution of the PL biarticular myogenic components remained <20% throughout the immobilization period. The ratio of the myofiber CSA of the immobilized to that of the sham-operated knees was significantly lower at 16 weeks after surgery than at 1 week after surgery only in the hamstrings.

**Conclusions:**

The relative contribution of the PT and PL components to myogenic contracture did not significantly change during the experimental period. However, the ratio of hamstrings CSAs to the sham side was larger than the ratio of medial gastrocnemius CSAs to the sham side after complete atrophy because of immobilization.

## Background

The normal range of joint motion (ROM) is maintained by repeated daily movements. The normal ROM is difficult to restore once lost [1], and immobilization is a major cause of joint contracture. Physical therapy and surgical release are used to prevent and treat joint contracture [2-4]. Knee flexion contracture can be surgically treated by posterior soft tissue release such as hamstring lengthening, proximal gastrocnemius release, and posterior capsule release [2]. Studies on animal muscles have shown that passive extensibility depends on the size and length of the muscle fibers and the amount and arrangement of connective tissue in the muscle belly [5-7].

Contracture can occur when a muscle undergoes disuse, as in the case of limb immobilization [8]. The technique of fixation of muscles at abnormally short lengths can be used to study muscle atrophy [7,9,10]. In a previous study, the components of joint contracture after immobilization were classified into arthrogenic and myogenic components [11]. However, to the best of our knowledge, no study has investigated the influence of myogenic components in detail. The low activity of immobilized muscles leads to muscle atrophy [9,12]. Muscle atrophy is caused by loss of tissue protein because of decreased synthesis and increased degradation [9,13], an increase in the amount of intramuscular connective tissue [14,15], and the arrangement of collagen fibrils in the endomysium [14,16,17]. Muscle atrophy from immobilization in shortened position is characterized by loss of muscle mass [18] and decrease in the size or cross-sectional area (CSA) of myofibers [19,20] and muscle length [5]. Few studies have addressed the relationship between muscle limitation of ROM and the changes in CSA specifically in biarticular muscles after atrophy because of immobilization.

The lower limb has three joints: the hip, knee, and ankle. The lower limb has many sites of muscle attachment. Biarticular muscles, in particular, are structurally coupled to the joints [21] and contribute strongly to myogenic restriction of ROM [22]. In the clinical situation, the hamstrings and gastrocnemius are often manipulated to prevent the progressive contracture or muscle atrophy when joint ROM is restricted [2,22-24]. The major focus of previous reports related to muscle CSAs and immobilization have been on monoarticular muscle [12,15,25]. However, the effect of joint immobilization on the myostatic properties and CSAs changes of biarticular muscles in the rat has not been extensively studied.

Our objective in this study was twofold: (1) to identify the relationship between biarticular muscles of the post thigh (PT; those muscles that cross the hip and knee joints) and those of the post leg (PL; those muscles that cross the knee and ankle joints) with the limitation of knee ROM after surgical immobilization and (2) to identify the relationship between the limitation of ROM at each phase of the contracture process and the changes in the CSA early (partial atrophy) and late (complete atrophy) after surgical immobilization.

## Methods

### Sample and surgical procedure

**Figure 1:**
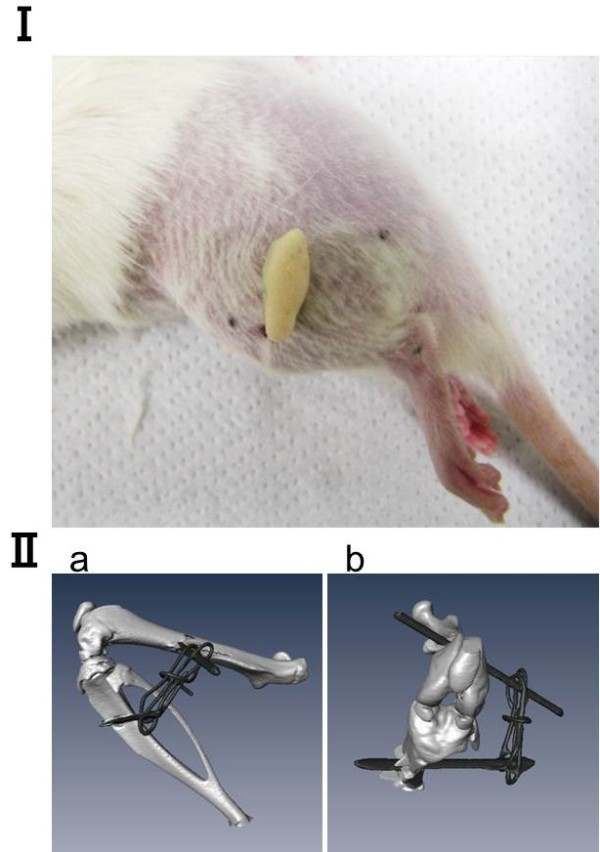
**Rat knee joint immobilized by external flexion with wire and resin. (I)** Photograph of the lateral view. **(II)** Micro-computed tomography analysis of the bone and wires from the **(II-a)** lateral and **(II-b)** front.

The experimental design for this study was approved by the College Animal Research Committee of Kyoto University (permission number: 12597). We used a total of forty 8-week-old male Wistar rats weighing 178–213 g randomly allocated in groups of 5 experimental and 3 control to the following five time points: 1, 2, 4, 8 and 16 weeks after surgical immobilization. The left hind limb of each experimental animal was immobilized with an external fixator consisting of wire and resin. Under sodium Nembutal anesthesia and sterile conditions, Kirschner wires were screwed into the femur and the tibia and fixed with wire and resin to maintain knee flexion of approximately 140° ± 5° (Figure 1). The flexed knee was thus rendered immobile. The right knee joint was subjected to sham surgery and was freely movable postoperatively. The control groups were included to control for the effect of age. The animals in each 1- and 16-weeks post-surgical experimental and control groups were used for morphological analysis. A high-resolution micro-CT scanner (SMX-100CT, Shimazu, Japan) was used to check the insertion site of the wires and view the immobilized leg. The leg was scanned from the ankle joint to the hip joint at the end of immobilization, i.e., when myotomy was performed. A bone and wire image was generated from the 3D image data sets by using a software (Amira 5.4, Visage, Germany).

All animals were housed in groups of 2 or 3 in plastic cages in an environmentally controlled room and fed rat food and water *ad libitum*.

### ROM analysis

At the end of the immobilization period, the animals were sacrificed under anesthesia with Nembutal and exsanguination. The wire and resin were removed from the joint and ROM analysis was performed. The macroscopic images were photographed with a digital camera (EX-V7, Casio, Japan) from the upper side. Thereafter the ROM was calculated using the Image J software package (National Institutes of Health, USA). The measurement method used was adopted from a previous study [25] and was slightly modified. We used a force gauge (DS2 series, Imada, Japan) to ensure that the direction and tension applied were the same as those in the original method. The ROM was defined as the angle (0° to 180°) between a straight line connecting the greater trochanter and the caput fibulae to a line connecting the caput fibulae and lateral malleolus with the hip joint at 90° of flexion. The maximum knee extension was defined as an extension of 180°. As the knee joint was extended passively for measurement, the trunk and pelvis were held manually to prevent the animal’s body from sliding forward. As previously described [26], the probe of the force gauges used to measure the ROM was fitted to the distal part of the ankle and then the strings were pulled with a tension of 0.49 N in the direction perpendicular to the trunk to extend the knee joint. The maximum knee extension was measured three times: (1) when the limb was intact, (2) after removal of the skin and PT from the hind limb, and (3) after removal of the PL from the lower leg. The muscles were removed beginning with their distal attachments. Distal incisions of PT that broadly attached to the front of the tibia were made from the tibia to their origins (proximal attachments) at the ischial tuberosity. Distal incisions of the PL were made from the distal Achilles tendon, which adhered to the calcaneus, to their origins (proximal attachments) on the femur. The incisions were made with caution to avoid damage to any additional muscles.

### Calculation of the arthrogenic and the PT and PL biarticular myogenic components of contracture

We evaluated myogenic contracture caused by the biarticular muscles of the leg, including their tendons and fascia, and arthrogenic contracture caused by the articular structures (bone, cartilage, synovium/subsynovium, capsule, and ligaments); myogenic and arthrogenic contractures were calculated with the use of the ROM measurements in the methods prescribed by Trudel et al [11]. The total contractures were independently calculated by the use of the ROM of experimental groups compared with those of sham-operated at each measurement time point to normalize. Myogenic contractures were further classified as those caused by PT or PL components. PT components were those crossing the hip and knee joints and PL components were those crossing the knee and ankle joints. The each of PT or PL components of myogenic contracture obtained after muscle detachment was used to estimate the each of biarticular muscles myogenic contracture. The each of PT or PL components of myogenic contracture were calculated by compared with the same sample.

The formulas used were as follows: (1) PT components = (ROM after biarticular myotomy at post thigh [immobilized group] – ROM before biarticular myotomy at post thigh [immobilized group]); (2) PL components = (ROM after biarticular myotomy at post limb [immobilized group] - ROM before biarticular myotomy at post limb [immobilized group]).

### Morphological analysis

After myotomy of the biarticular muscles, we macroscopically confirmed the difference between the PT and PL. In the control group, the macroscopic images were photographed with a digital camera from the upper side. Serial cross sections of 10 μm were made using a cryostat (at -15°C); the portions from the middle part of the muscle belly of the bilateral hamstring and medial gastrocnemius muscles (CM1900, Leica, Germany) were stained with hematoxylin and eosin (H-E) for histological observation. Photographs (magnification: ×100) were taken of each section with a microscopy camera (ECLIPSE 80i, Nikon, Japan). A measuring field was defined over the entire muscle cross-section. The CSAs of at least 100 randomly selected muscle fibers were measured using the Image J software program. Thereafter the mean fiber size of each muscle CSAs were calculated. Representative sections data are shown.

### Statistical analysis

All data are shown as mean ± standard deviation (SD). The software program SPSS Statistics (IBM, USA) was used for statistical analysis. Differences in the ROM between the experimental and sham or control groups at each time point and differences in the CSAs of the hamstring and gastrocnemius muscles between the experimental periods were assessed using Student’s *t*-test. The significant difference between the experimental and the sham or control groups at the same time point was measured at 95% CI not overlapping zero. One-way analysis of variance (ANOVA) and the Tukey-Kramer test were performed to examine differences in ROM among the time points.

## Results

All of the rats survived, gained weight, and remained active throughout the experimental period. Neither prolonged edema nor acute inflammation was observed in any animal.

### ROM analysis and relationship between the myogenic and arthrogenic components of contracture

**Figure 2:**
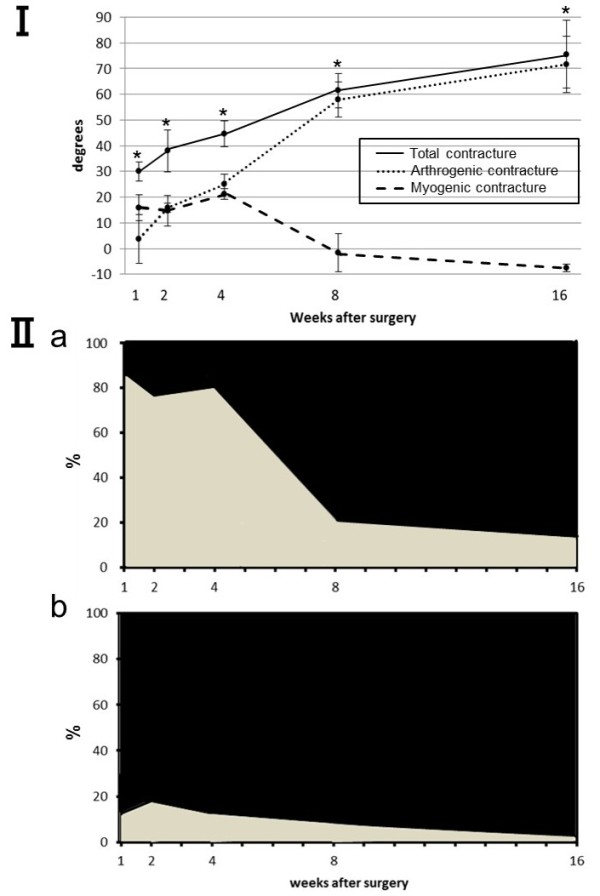
**The myogenic and arthrogenic contractures in immobilized knee joints over time. (I)** Results in degrees. As the duration of immobilization increased, the myogenic contracture decreased and the arthrogenic contracture increased. Values are presented as mean ± SD. ^*^Significant difference in the restriction of extension ROM between the immobilized and sham knees. ^*^P < 0.05. **(II)** Results as presented as the percent contributions of the biarticular muscles to the total restriction: **(II-a)** results for the PT, PT components shown in gray; results for other components shown in black; **(II-b)** results for the PL, PL components shown in gray; results for other components shown in black. The PT contribution peaked after the first week and decreased thereafter. The peak contribution of the PL was significantly less than that of the PT.

The loss of extensional ROM was fairly similar between sham-operated and control knees except at the 4-week time point. Four week after surgery, the extensional ROM in the sham knees was smaller than that in the control animals (*P* = 0.04, data not shown). Knee extension was significantly restricted in all experimentally immobilized knee joints as compared with that in sham-operated knee joints, at the same time point (*P* < 0.05; Figure 2 I; Table 1). The myogenic contracture peaked 4 weeks after surgery and decreased thereafter. The arthrogenic contracture progressed especially rapidly between postoperative weeks 4 and 8. The myogenic contracture in the immobilized knees was significantly lower than the arthrogenic contracture at the 8- and 16-week time points (*P* < 0.01; Table 1).

**Table 1:** Arthrogenic and myogenic contracture contributions to the total limitation of extension ROM

**After immobilization**	**1 week**	**2 weeks**	**4 weeks**	**8 weeks**	**16 weeks**
Total contracture	30.0 ± 4.1^†^	38.0 ± 9.0^†^	44.5 ± 5.5^†^	61.4 ± 7.5^†^	75.5 ± 14.8^†^
Myogenic contracture	15.9 ± 5.6	14.7 ± 6.5	21.0 ± 2.2	-1.6 ± 8.2^‡^	-7.5 ± 1.5^‡^
Arthrogenic contracture	3.7 ± 10.5	15.7 ± 1.9	25.0 ± 4.1	57.9 ± 7.6^‡^	71.5 ± 12.2^‡^

Values are given as mean ± SD. All data are presented in (°).

^†^Indicates significant difference was found when the given data were compared with sham data in the same time point.

^‡^Indicate significant difference was found when the given data were compared with the myogenic and arthrogenic contracture in the same time point.

### PT and PL components involved in the limitation of extension angular displacement

**Figure 3:**
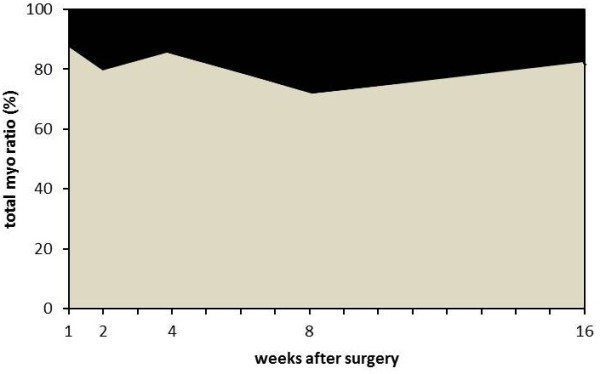
**The percent contributions of the PT and PL to myogenic contracture.** PL components shown in black; PT components shown in gray. The relative contributions of the PT and PL to myogenic contracture did not change throughout the experimental period.

In the immobilized knees, the relative contribution of the PT components to the total restriction reached 82.4 ± 15.6% after the first postoperative week but decreased thereafter and was significantly low 8 and 16 weeks after surgery (*P* < 0.01; Figure 2 II-a, Table 2). The relative contribution of the PL components reached 18 ± 14.8% after 2 weeks and throughout the remainder of the immobilization period (Figure 2 II-b, Table 2). However, the ratio of the PT and PL contributions to the myogenic contracture did not change throughout the experimental period (R^2^ = 0.006; Figure 3). In the sham-operated knees, the relative contribution of the PT components to the myogenic contracture was >76% and almost plateaued throughout the experimental period similar to the immobilized knees.

**Table 2:** PT and PL of myogenic components in total extension ROM after knee joint immobilization

**After immobilization**	**1 week**	**2 weeks**	**4 weeks**	**8 weeks**	**16 weeks**
Total contracture (°)	30.0 ± 4.1	38.0 ± 9.0	44.5 ± 5.5	61.4 ± 7.5	75.5 ± 14.8
PT components (°) ratio of total (%)	25.6 ± 2.7	28.3 ± 7.5	35.1 ± 4.7	12.3 ± 5.2	10.0 ± 1.9
	82.4 ± 15.6	70.2 ± 20.0	66.5 ± 11.7	20.9 ± 10.6^†^	13.9 ± 4.6^†^
PL components (°) ratio of total (%)	3.9 ± 3.9	7.0 ± 5.3	5.6 ± 3.1	5.3 ± 3.9	2.1 ± 0.9
	12.5 ± 13.5	18.0 ± 14.8	12.7 ± 7.2	8.6 ± 6.8	2.7 ± 1.3

Values are given as mean ± SD.

^†^Indicates significant difference was found when the given data were compared with the data for 1, 2 and 4 weeks after surgery.

### Morphological appearance and CSAs of the muscle fibers

**Figure 4:**
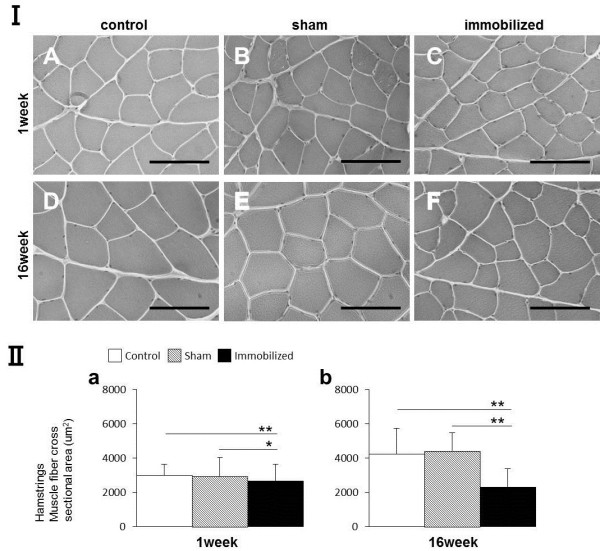
**Morphological analysis and myofiber CSAs measurements (μm**^**2**^**) of the hamstring muscle.** (I) The CSA of the hamstring muscles of control, sham-operated, and immobilized knees after 1 and 16 weeks. **(A, D)** Control group. **(B, E)** Sham-operated knees. **(C, F)** Immobilized knees. Scale bars represent 100 μm (microscope magnification: ×400). **(II-a, b)** Measurement of the CSA (μm^2^) of the hamstring muscles (only one displayed). Values are presented as mean ± SD; ^*^P < 0.05; ^**^P < 0.001. The CSA in the immobilized knees was lower than that in the control or sham knees at both time points.

**Figure 5:**
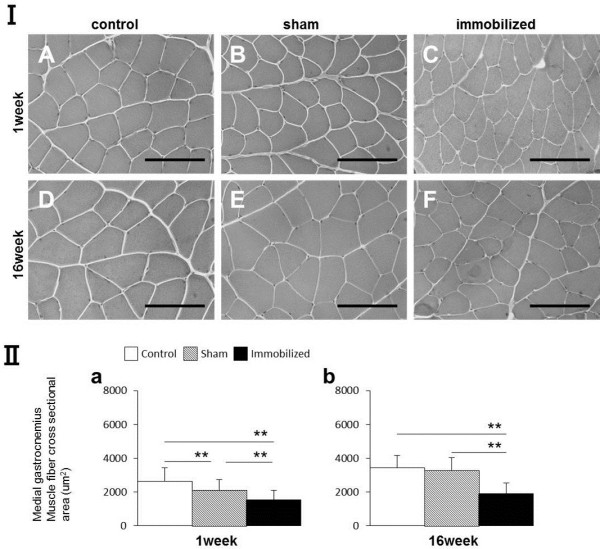
**Morphological analysis and myofiber CSA measurements (μm**^**2**^**) of the medial gastrocnemius muscle. (I)** The CSAs of the medial gastrocnemius muscle from the control, sham-operated, and immobilized knees after 1 and 16 weeks. **(A, D)** Control group. **(B, E)** Sham-operated knees. **(C, F)** Immobilized knees. Scale bars represent 100 μm (microscope magnification: ×400). **(II-a, b)** Measurement of the CSA (μm^2^) of the medial gastrocnemius muscle (only one displayed). Values are presented as mean ± SD; ^**^P < 0.001. The CSA in the immobilized knees was lower than that of the control or sham knees at both time points.

**Figure 6:**
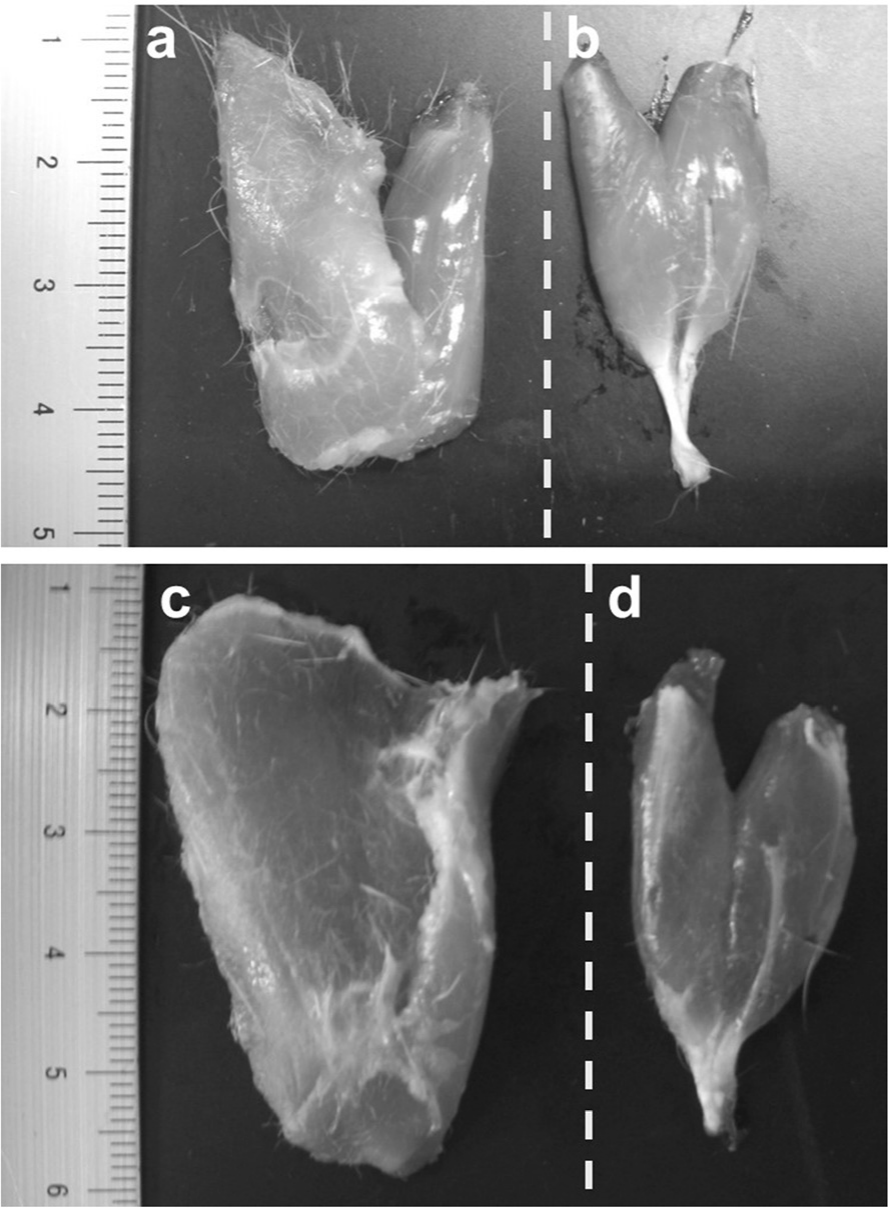
**Macroscopic observation of PT and PL of the control group after 1 and 16 weeks. (a)** PT at the 1-week, **(b)** PL at 1 week, **(c)** PT at 16 weeks, **(d)** PL at 16 weeks. The dotted line shows the medial side. The muscle length of PT (including the hamstrings) were longer than that of PL (including the gastrocnemius).

In the control group, myofiber CSAs of the hamstring and medial gastrocnemius muscles were larger after 16 weeks than 1 week after surgery (Figure 4 I-A, D; Figure 5 I-A, D). In macroscopic observation, the muscle length of hamstrings was longer than that of gastrocnemius in control group (Figure 6).

#### The CSAs of hamstring muscle fiber

After 1 and 16 postoperative weeks, the CSAs of the hamstring of the immobilized knees were found to be significantly smaller than those of the sham-operated or the control group (^*^P < 0.05; Figure 4 II-a, ^**^P < 0.001; Figure 4 II-a,b). These trends were similar to all samples.

#### The CSAs of medial gastrocnemius muscle fiber

After 1 and 16 postoperative weeks, the CSAs of the medial gastrocnemius muscles of the immobilized knees were significantly smaller than those of the sham-operated or control group. One week after surgery, the CSAs of the medial gastrocnemius muscles of the sham-operated knees were significantly smaller than those of the control group (^**^P < 0.001; Figure 5 II-a,b). These trends were similar to all samples.

### Comparison of the hamstring and gastrocnemius CSAs

**Figure 7:**
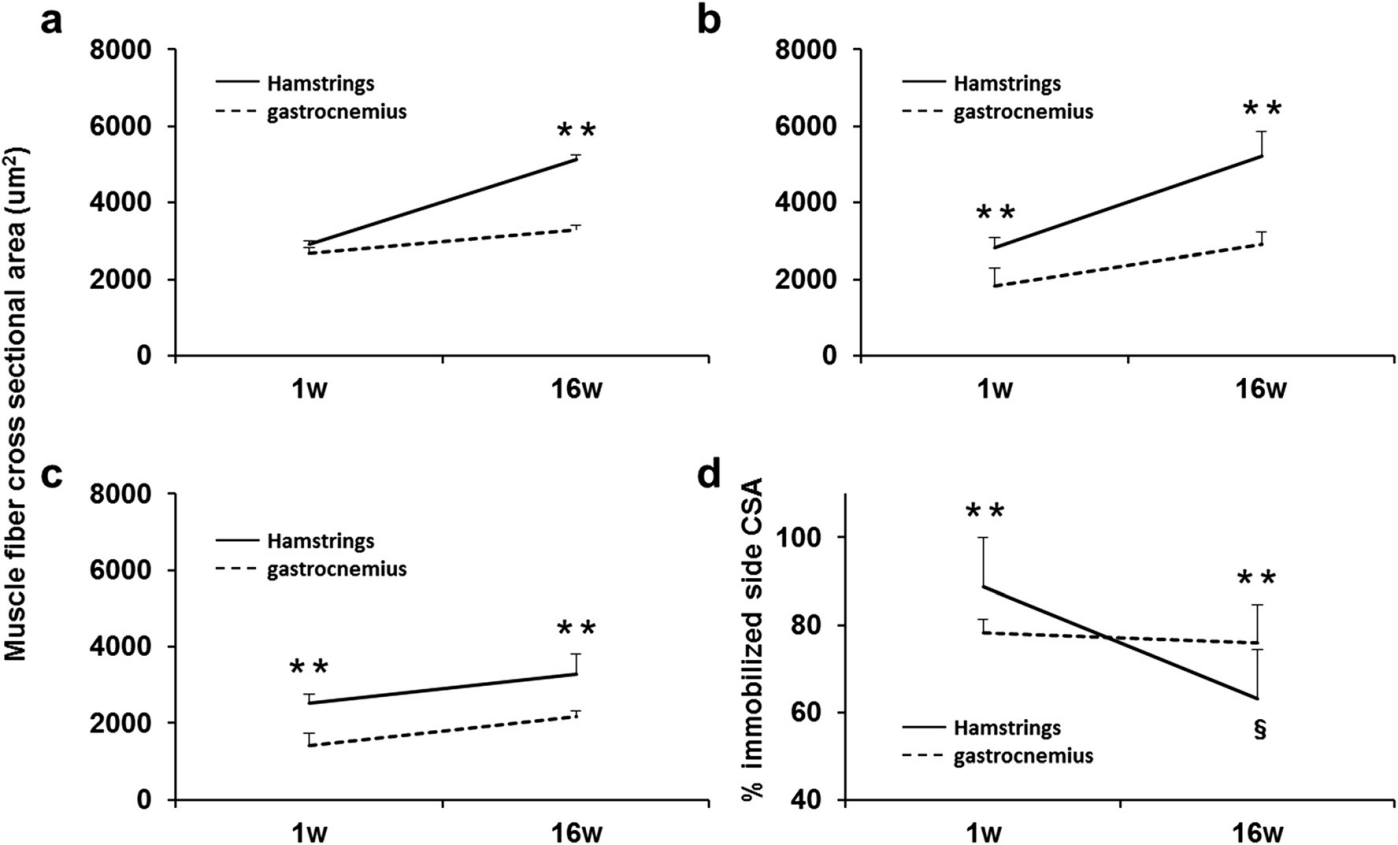
**Changes in the CSAs and comparison of hamstring and gastrocnemius cross sectional areas during the experimental periods.** Cross-sectional area (μm^2^) of the control group **(a)**, sham-operated group **(b)**, and immobilized group **(c)**. The ratio of the immobilized side CSA to the sham side CSA (%) **(d)**. Values are presented as mean + SD; **, hamstring vs gastrocnemius at the same time point (*P* < 0.01); §, 1 week vs 16 weeks of hamstring (*P* < 0.01). Significant differences were found the CSAs between the hamstring and the medial gastrocnemius muscles at each time point in the all groups, except for at 1 week in the control group. The ratio was significantly lower after 16 weeks than after 1 week for the hamstring but not for the gastrocnemius. The myofiber CSAs of the hamstring were larger than those of the gastrocnemius at each time point in all groups, except for at 1 week in control group.

In histologically, the myofiber CSAs of the hamstring were larger than those of the gastrocnemius muscles at each time point in the sham and immobilized knees (Figures 4, 5 I). The CSAs of the hamstring were significantly larger than those of the medial gastrocnemius muscles at each time point in the all groups (*P* < 0.01; Figure 7-a,b,c), except for at 1 week in the control group (Figure 7-a). The CSAs of each muscles at 16 weeks were significantly larger than those at 1 week in the all groups (*P* < 0.01), except for those of the gastrocnemius in the control group (*P* < 0.05). The ratio of the hamstring CSAs of the immobilized knee to those of the sham-operated knee significantly decreased between postoperative weeks 1 and 16 (*P* < 0.05; Figure 7-d); however, the ratio for the gastrocnemius did not significantly change.

## Discussion

In the present study, the myogenic contracture in the immobilized knee have significantly deceased than arthrogenic contracture after 8 week time point. However, the relative contribution of the PT and PL components to the myogenic contracture did not significantly change throughout the experimental period. This results suggested that the PT components had a greater impact than the PL components on the muscle contracture through the experimental period. Immobilization in a shortened position produced the most extreme muscle atrophy due to significant shortening of the fibers and reduction in the CSA [18-20]. It suggested that both of PT and PL muscles have got atrophy and they lead muscle ROM limitation.

In the immobilized knees, the relative contributions of the PT and PL to the total contracture and the myogenic contracture exhibited different tendencies between the early and late of experimental period. The ratio of the CSAs of the hamstring of the immobilized knees to those of the sham side was significantly smaller after 16 weeks than after 1 week of immobilization. However, the CSAs of the gastrocnemius did not change remarkably at both of the early and late time point. In the present study, immature rats were treated. Muscle fiber diameter increases dramatically during early growth animals. The peak change in the increased diameter and fiber number differs for each of muscle [14]. Owing to aging, the compensatory hypertrophy of some fibers brought about by the normal increase in body weight resulted in increased load on the muscle [27]. The pathways that contribute to the increase in apoptosis observed in acutely atrophying muscle differ strikingly according to the age [28] and muscle type [4,14,20].

In this study, the myofiber CSAs were larger in the hamstrings than in the gastrocnemius muscle in all groups. We confirmed that the muscle length of hamstrings was longer than that of gastrocnemius in the control group. The muscle mass was calculated by multiplying the CSA and the muscle length [29]. A previous study on the muscle architecture of the rat hind limb, showed that the muscle fiber length of the hamstrings was larger than that of the gastrocnemius [30]; this holds true for humans too [31]. Muscle mass can influence the self-inertia of a joint [32]. Passive extensibility is influenced by the size (mass) and length of muscle fibers [5,33] and the amount and arrangement of the connective tissues of the muscle belly [5]. This means that the muscle length and CSAs of the PT (including the hamstrings) were larger than those of the PL (including the gastrocnemius), suggesting that PT was more effective than PL in restricting the muscle extensibility because of increased collagen fibers in its connective tissues.

In addition, one potential explanation is that the difference in the contributions of the PT and PL could also be attributed to the difference in their lever arms, i.e., the distance between the point at which a force is applied and the axis. Notably, the lever arm of a muscle depends on the distance between its attachment points. According to one study on the muscles of the frog legs, the relationship between the moment arm and sarcomere length related to each proximal and distal joint angle [6]. However the length of the moment arm of the biarticular muscle is not always equal to the one located distal or proximal to it [34]. The PL components in this study originated very close to the axes of rotation of the knee and ankle, their effective lever arms very small. However, the PT distally and broadly extended on the front of the tibia, so their lever arms were larger than those of the PL, a result suggesting that the PT lengthens more than the PL during extension. Passive stretch and isometric tension are suggested to stimulate protein synthesis [9] and thus increase extensibility [22,32]. In this study, the PT components contributed more than the PL components to myogenic contracture, suggesting that knee joint immobilization restricted the extension of the PT more than it did of the PL, thereby promoting pronounced PT atrophy.

This relationship and other changes in the biarticular muscles of the legs after immobilization are worthwhile topics for future research.

### Limitations

In this study, we measured the ROM of the knee joint adjacent to a freely mobile ankle joint. The traction string was attached to the calcaneus, but the influence of the ankle joint may not have been fully evaluated. Passive plantar flexion of the talocrural joint would have allowed us to better evaluate the collective contribution of the PL to the myogenic contracture. There were technical limitations associated with the arthrometer. For quantitative evaluation, we used a force gauge with the moment arm manually held, but manually applied power generates an inaccurate torque. Nevertheless, the loss of angle and the gradual increase in arthrogenic contracture after 4, 8, and 16 weeks in our study were similar to those mentioned in a previous report [11], suggesting that our method was reasonably accurate. With regard to study design, it is advisable to use a large sample size and to choose sample by randomly detaching each biarticular muscle.

## Conclusions

This report showed that the relative contribution of the PT and PL components to myogenic contracture did not significantly change during the experimental period. However, the ratio of hamstring CSAs to the sham side was larger than the ratio of medial gastrocnemius CSAs to the sham side after complete atrophy because of immobilization. In conclusion, our findings suggest that the contribution of the biarticular muscles to the limitation in the knee extension ROM after knee joint immobilization was predominantly caused by PT rather than PL.

## Abbreviations

PT: Biarticular muscles at the post thigh, which means behind the thigh; PL: Biarticular muscles at the post leg, which means behind the leg; ROM: Range of joint motion; CSA: Cross-sectional area.

## Competing interests

The authors declare that they have no competing interests.

## Authors’ contributions

MN, TA, AI, HI, SY, JT, XZ, HA, HK carried out conception or design of the study and were involved in the analysis and interpretation of the data. MN, TA, AI, HI, SY, JT, HK were involved in preparing the draft and conducting the statistical analysis. MN, TA, and HK approved the final version to be published. HK is the laboratory chair and obtained the funding. MN carried out the collection and assembly of data. All authors read and approved the final manuscript.

## Pre-publication history

The pre-publication history for this paper can be accessed here:

http://www.biomedcentral.com/1471-2474/15/224/prepub
